# Supplementary figures and images for: Impact of labor characteristics on maternal and neonatal outcomes of labor: A machine-learning model
Source: PLoS One. 2022 Aug 22;17(8):e0273178. doi: 10.1371/journal.pone.0273178 (PMC9394788; doi:10.1371/journal.pone.0273178)

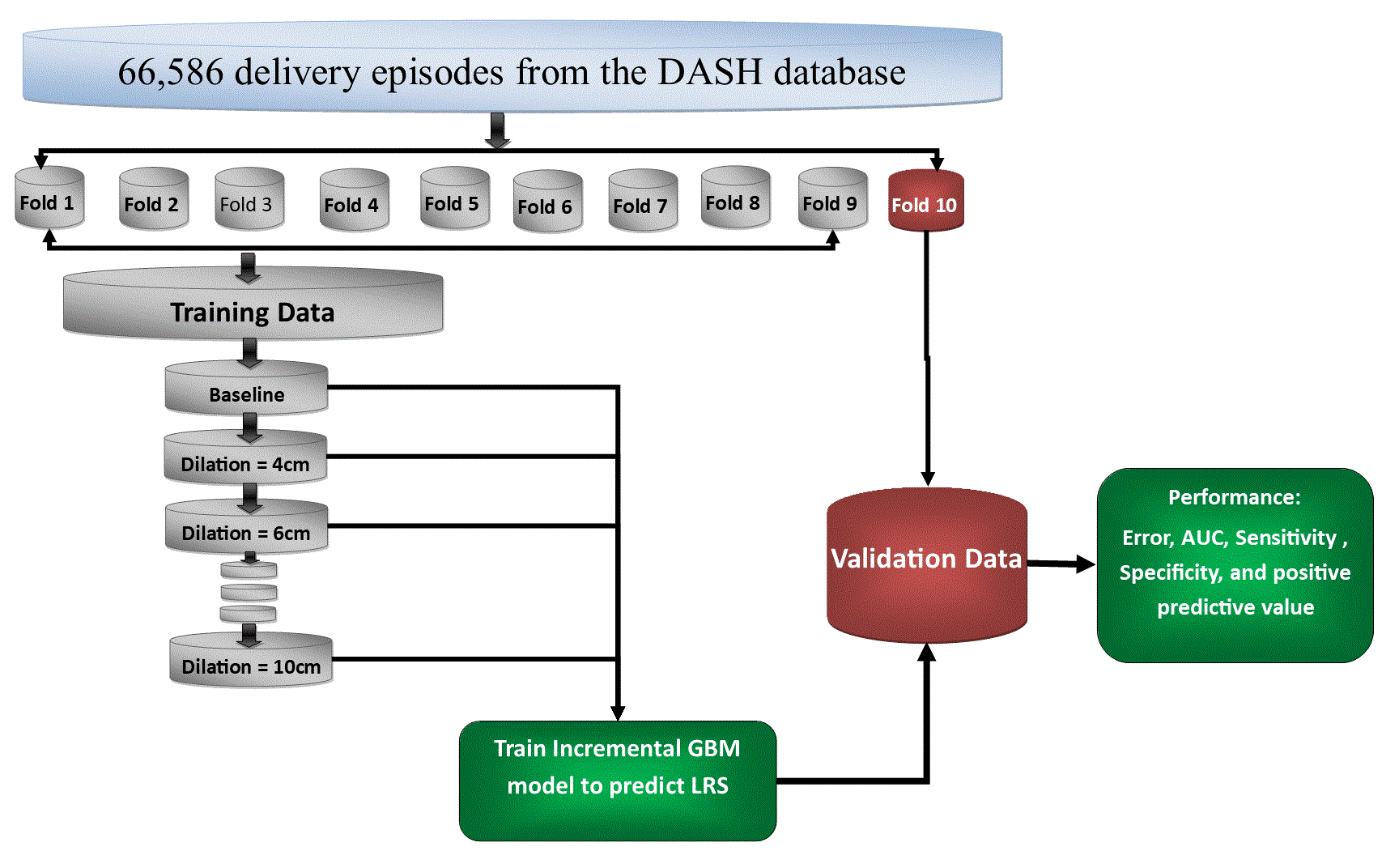

Supplement: S1 Fig — Each model (except baseline model) uses labor risk score (LRS) predictions from the previous model. Data were randomly divided into 10 equal and independent parts: The model was trained on 9 folds and validated on the last fold. The procedure was repeated until each fold was used once for validation. At each step, optimal tuning parameters of the model were selected, and performance was evaluated on the validation fold. Overall iterative process was repeated 10 times and performance results were averaged. (GIF) [file pone.0273178.s001.gif]

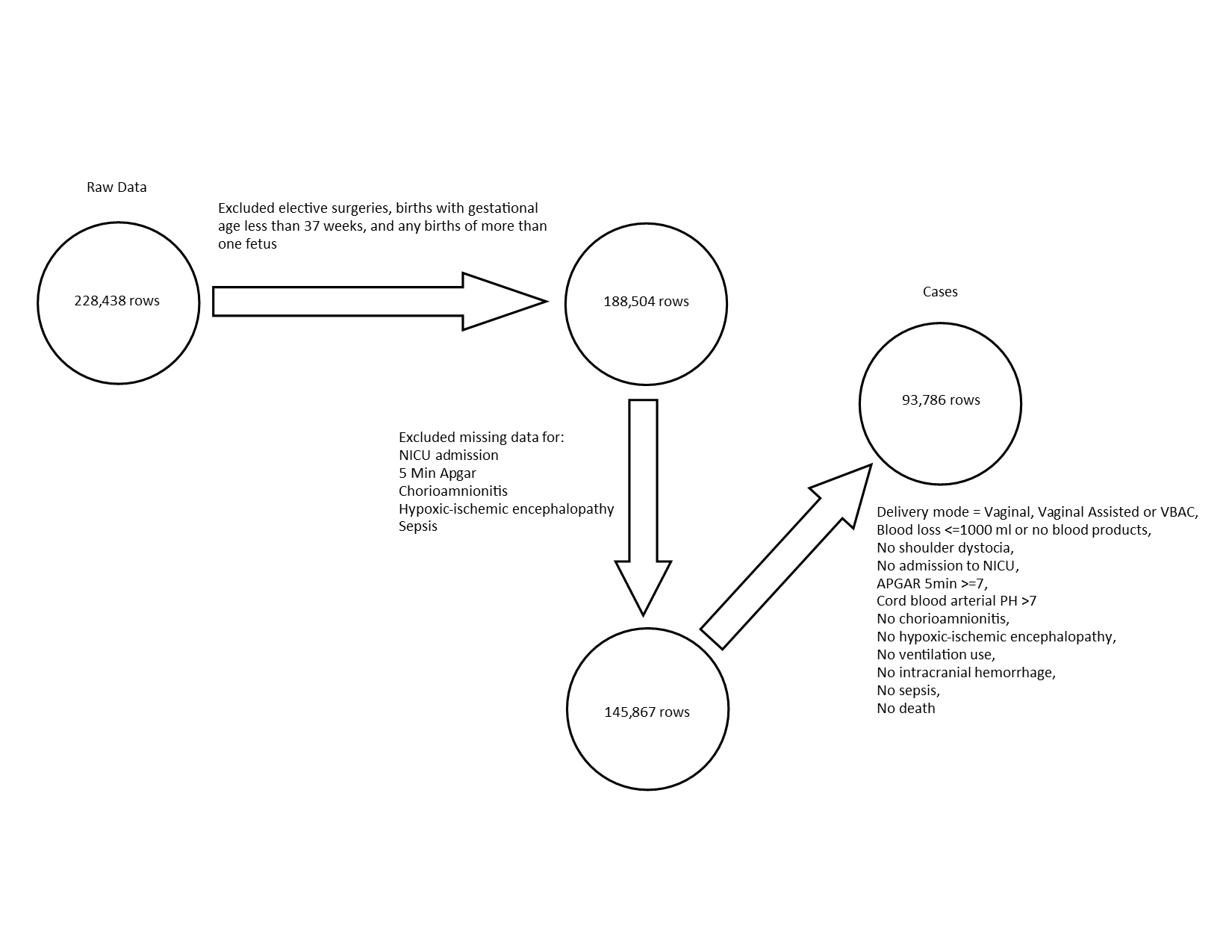

Supplement: S2 Fig — (PNG) [file pone.0273178.s002.png]

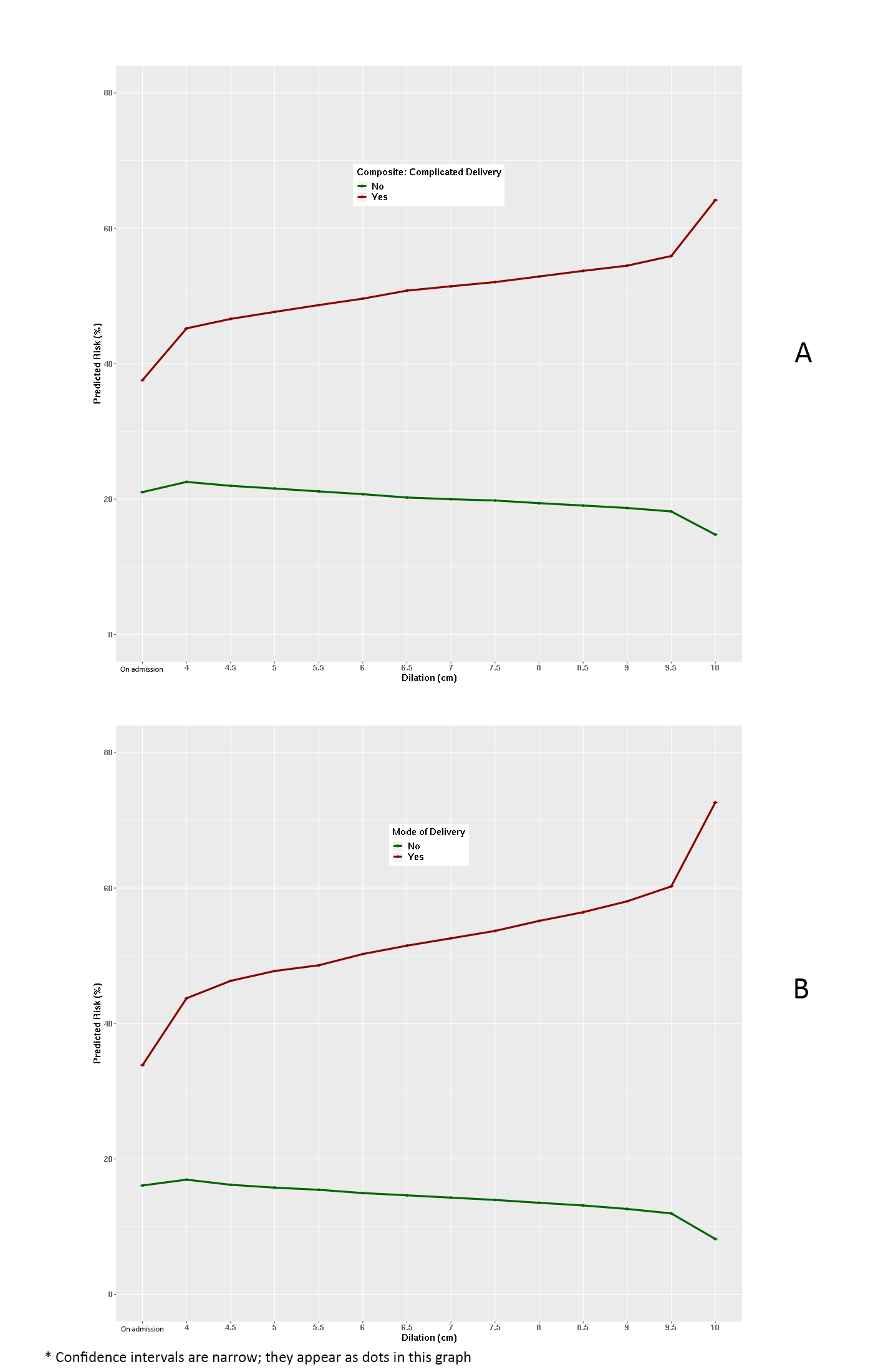

Supplement: S3 Fig — A, Women with unfavourable (red line) versus favourable (green line) composite labor outcome. B, Women who had cesarean delivery (red line) versus vaginal delivery (green line). (PNG) [file pone.0273178.s003.png]
