# Supplementary material for: Impact of labor characteristics on maternal and neonatal outcomes of labor: A machine-learning model
Source: PLoS One. 2022 Aug 22;17(8):e0273178. doi: 10.1371/journal.pone.0273178 (PMC9394788; doi:10.1371/journal.pone.0273178)
Supplement: S1 Table — (DOCX) [file pone.0273178.s004.docx]

| **Outcome** | **Number (%)** |
| --- | --- |
| Unfavorable composite outcome | 14439 (21.7%) |
| Intrapartum Cesarean delivery | 10466 (15.7%) |
| Admission to neonatal intensive care unit | 3743 (5.6%) |
| APGAR score < 7 at 5 minutes | 510 (0.8%) |
| Umbilical arterial pH < 7.00 | 108 (0.2%) |
| Intraamniotic infection | 2395 (3.6%) |
| Hypoxic ischemic encephalopathy | 5 (0.00%) |
| Need for neonatal ventilation | 269 (0.4%) |
| Neonatal intracranial hemorrhage | 43 (0.1%) |
| Neonatal sepsis | 880 (1.3%) |
| Neonatal death | 49 (0.1%) |

Table S1: Unfavorable labor outcomes among study population
